# Molecular Profiling of DNA Methylation and Alternative Splicing of Genes in Skeletal Muscle of Obese Rabbits

**DOI:** 10.3390/cimb43030110

**Published:** 2021-10-11

**Authors:** Yanhong Li, Jie Wang, Mauricio A. Elzo, Huimei Fan, Kun Du, Siqi Xia, Jiahao Shao, Tianfu Lai, Shenqiang Hu, Xianbo Jia, Songjia Lai

**Affiliations:** 1 College of Animal Science and Technology, Sichuan Agricultural University, Chengdu 611130, China; lyh81236718@163.com (Y.L.); wjie68@163.com (J.W.); fanhuimei1998@163.com (H.F.); Dukun1672@163.com (K.D.); xiasiqi2020@163.com (S.X.); Shao1997@163.com (J.S.); ltf970329@163.com (T.L.); shenqianghu@gmail.com (S.H.); jaxb369@sicau.edu.cn (X.J.); 2 Department of Animal Sciences, University of Florida, Gainesville, FL 32611, USA; maelzo@ufl.edu

**Keywords:** DNA methylation, alternative splicing, metabolic network, skeletal muscle, rabbit

## Abstract

DNA methylation and the alternative splicing of precursor messenger RNAs (pre-mRNAs) are two important genetic modification mechanisms. However, both are currently uncharacterized in the muscle metabolism of rabbits. Thus, we constructed the Tianfu black rabbit obesity model (obese rabbits fed with a 10% high-fat diet and control rabbits from 35 days to 70 days) and collected the skeletal muscle samples from the two groups for Genome methylation sequencing and RNA sequencing. DNA methylation data showed that the promoter regions of 599 genes and gene body region of 2522 genes had significantly differential methylation rates between the two groups, of which 288 genes had differential methylation rates in promoter and gene body regions. Analysis of alternative splicing showed 555 genes involved in exon skipping (ES) patterns, and 15 genes existed in differential methylation regions. Network analysis showed that 20 hub genes were associated with ubiquitinated protein degradation, muscle development pathways, and skeletal muscle energy metabolism. Our findings suggest that the two types of genetic modification have potential regulatory effects on skeletal muscle development and provide a basis for further mechanistic studies in the rabbit.

## 1. Introduction

The growth and development of muscle are closely related to nutrition, heredity, gender, and age and involve a variety of metabolic mechanisms. Studies have shown that the excessive accumulation of fat in obese humans can damage the AMPK signal pathway [1] and cause disorders in the microvascular function of skeletal muscle, leading to decreased insulin sensitivity [2]. Compared with those in normal mice, the excessive accumulations of visceral fat in obese mice cause the disordered secretion of adipokines, inflammatory reactions, abnormal distributions of skeletal muscle bundles, and muscle atrophy [3]. This phenomenon indicates that fat and muscle have a synergistic effect. Rabbit muscles have low cholesterol and high levels of unsaturated fatty acids. Simultaneously, rabbit meat has high nutritional value and contains a high level of proteins and low levels of fat and cholesterol. Therefore, rabbit meat is regarded as ideal for people with obesity and cardiovascular diseases [4]. However, the molecular mechanisms involved in the development and metabolism of the rabbit skeletal muscle are unclear.

DNA methylation is a chemical modification process in which a specific base in a DNA sequence is covalently bonded to form a methyl group with a methyl donor of S-adenosyl methionine (SAM) under the catalysis of DNA methyltransferases (DNMTs) [5]. DNA methylation is an important epigenetic mechanism, and most studies showed that changes in DNA methylation can influence energy metabolism in the muscle [6,7,8]. Studies indicated that differentially methylated CpG sites in muscle tissues vary with age and that gene body methylation sites are closely related to gene expression, influencing muscle development [9,10,11]. Muscle development is often accompanied by variation in energy metabolism. Different energy states can cause different development patterns in muscle. High-fat diets can cause abnormal energy metabolism and change the methylation status of genes in muscle. Short-term high-fat diets can change the level of DNA methylation in the young skeletal muscle, involving the expression of genes from the inflammatory signaling pathway [12]. Conversely, birth weight probably influences muscular methylation and the metabolism of high-fat diets. Under the condition of a high-fat diet, people with normal birth weight can produce a large number of methylation site differences than those with low birth weight [13]. In addition, the abnormal DNA methylation in skeletal muscle can be induced by different diet energy sources and may be one of the important factors leading to metabolic diseases, such as obesity and type II diabetes [14]. These studies demonstrated that energy metabolism can cause changes in the pattern of tissue methylation modification, suggesting that the same methylation modification mechanisms likely exist in the rabbit skeletal muscle.

DNA methylation sites exist in different functional regions of genes and can regulate the expression levels of regulatory genes. Studies showed that the promoter and gene body region methylation mechanisms play an important role in regulating gene expression and function [15,16]. Exercise can change the energy metabolism of muscles and decrease gene promoter methylation in the skeletal muscle of older people. Furthermore, the hypomethylation of gene promoters is associated with oxidative stress response genes and muscle development proteins, such as myosin, atrophic protein, and actin, thereby increasing the expression level of these genes and leading to increased skeletal muscle sensitivity to insulin and antioxidative stress capability [17]. Gene body methylation in the skeletal muscle of pigs increases with age is negatively correlated with gene expression and regulates the function of protein hydrolysis and degradation [18]. Gene promoter methylation directly affects the processes of muscle proliferation and differentiation, influencing the mRNA expression levels of myostatin (*MSTN*), stearoyl-CoA desaturase (*SCD*) gene, and fibroblast growth factor (FGF) 21 [19,20,21]. Hence, age differences and diets with different energy sources affect DNA methylation types in skeletal muscle, thereby increasing the accuracy of biological gene responses. The same methylation modification mechanisms likely exist in the rabbit skeletal muscle. 

Alternative splicing is an important genetic modification mechanism for transcript ing the gene into different forms of mRNA [22]. Alternative splicing includes seven kinds of alternative splicing forms, i.e., exon skipping (ES), retained intron (RI), mutually exclusive exon (MXE), alternative 5′ splice site (A5SS), alternative 3′ splice site (A3SS), alternative first exon (AFE), an alternative last exon (ALE). Human studies found that ES is the main alternative splicing form (35%), followed by variable 3′ (16%) and variable 5′ (15%) termini. Intron retention is the least frequent, accounting for only 1% of all alternative splicing events [23]. Previous studies showed a close relationship between gene methylation and alternative splicing, affecting a variety of important biological processes through genes interaction and their involved signal pathways [24,25,26]. DNA methylation can influence the structure of nuclear chromatin by interfering with transcription factors to silence genes [27]. In addition, the probability of CG methylation sites in exons is higher than that in introns, enhancing the recognition strength of alternative splicing signals and the coding of different splicing precursor mRNA forms and resulting in the translation of proteins with new functions [28,29]. Transcriptome sequencing shows that DNA methylation and alternative splicing differences exist in tissues and individuals, resulting in different effects on gene regions to adjust gene expression levels [30,31]. The genetic modification of genes is the key to forming various functions of the coding protein, suggesting involvement in a complex regulatory network. The metabolic pattern of the rabbit skeletal muscle may have a potential molecular regulatory network, which is closely related to genetic modification.

Therefore, this study aims to establish a high-fat diet-induced obese rabbit model and analyze the changes in DNA methylation and alternative splicing data in the rabbit skeletal muscle between the two groups. Subsequently, we have found that most of the genes have significant differences in variation between the two data sets, and genes with two genetic mechanisms of methylation and alternative splicing are our focus. Moreover, a potential metabolic regulatory network is constructed in the skeletal muscle to provide a basis for the genetic mechanism studies of rabbit meat quality and human metabolic diseases.

## 2. Materials and Methods

### 2.1. Construction of the High Fat Diet-Induced Obese Rabbit Model

Tianfu black rabbit breed was performed under a strictly systematic breeding process and had high meat quality with lower intramuscular fat than other rabbits [32]. Then, it can be treated as an ideal material for us to construct an obese model for investigating the metabolism differences in rabbit skeletal muscle. Female rabbits (*n* = 24) at about 35 days of age were selected from the Tianfu black rabbit colony in the teaching and research rabbit farm of Sichuan Agriculture University and randomly divided into two groups, a control group (CON−G; *n* = 12) and a high-fat diet-induced group (HFD−G; *n* = 12). The CON−G rabbits and HFD−G rabbits were fed with a commercial diet and a mixed diet composed of a commercial diet plus 10% pork lard, respectively. All rabbits were fed from 35 d to 70 d, and specific feed procedures were performed in accordance with our previous study [33]. All the rabbits adopted the same breeding management conditions and were vaccinated regularly. The three rabbits with significant body weight and characteristics of obesity were selected as HFD−G rabbits, while the three rabbits with normal weight and physiological characteristics were chosen for CON−G rabbits. All the six rabbits from the two groups were killed by air injection into the auricular vein and slaughtered to collect right *biceps femoris* muscle samples for the extraction of total RNA and DNA.

### 2.2. Total RNA Extraction and RNA-Sequencing

Total RNA was extracted from skeletal muscle samples (stored at −80 °C) in accordance with the TakaRa MiniBEST Universal RNA Extraction Kit instruction manual (TakaRa, Dalian, China). The concentration and purity of RNA were determined by using the NanoDrop 2000 Spectrophotometer (Thermo Fisher Scientific, Waltham, MA, USA). Three RNA samples from each group were respectively prepared for the construction of RNA libraries, using NEBNext^®^ Ultra™ Directional RNA Library Prep Kit for Illumina^®^ (NEB, Ipswich, MA, USA) according to its recommendations. Subsequently, all the RNA sequencing libraries were sequenced using the Illumina HiSeq X Ten platform. The raw reads needed to remove unqualified sequencing reads, including reads containing adapters, reads with a ratio of N greater than 5%, low-quality reads (the number of bases with a quality value of Q ≤ 10 accounts for more than 20% of the entire read), and rRNA reads. Data processing was performed by Chengdu Life Baseline S&T Co.Ltd. Clean reads with high quality were obtained after filtering and data quality control of raw data. The Hisat2 software v2.0.0 (http://ccb.jhu.edu/software/hisat2/downloads/, accessed on 7 January 2019) was used to match clean reads by comparing them with the reference genome sequence (GCF_000003625.3) to assess overall sequencing quality [34]. The parameters of the software were referred to in accordance with the step: --phred64 --sensitive --no-discordant --no-mixed -I 1 -× 1000. Gene quantitative analysis was carried out using the Kallisto software 0.43.0 through transcripts Per Million (TPM) and filtering out of genes (TPM < 1) in all samples [35]. 

### 2.3. DNA Extraction and Whole-Genome Bisulfite Sequencing (WGBS)

DNA samples from rabbit muscle tissues were respectively extracted using the QIAGEN DNA Kit (QIAGEN, Dusseldorf, Germany) in accordance with its manufactured guidelines. DNA concentration was determined using agarose gel electrophoresis and the NanoDrop 2000 Spectrophotometer. To better avoid inter-individual variability and preliminary discovery of important genes in skeletal muscle, the methylation sequencing samples of skeletal muscle tissue from each group were consistent with the above transcriptome sequencing muscle tissue samples. Simultaneously, the three DNA samples from each group were mixed and prepared for the construction of two libraries. The genomic DNA was broken by ultrasound into 300 bp fragments, which should be repaired at the end of each fragment, added with an A base at the 3′ end, and connected to the sequencing joint. Subsequently, the constructed libraries were treated with a bisulfite treatment, using the ZYMO EZ DNA Methylation-Gold kit (ZymoResearch, Los Angeles, USA), and the DNA fragments were amplified using specific amplification conditions. Finally, the bisulfite-treated libraries were constructed and sequenced using the Illumina Hiseq™ X Ten system [36]. 

### 2.4. Processing and Comparison of the Bisulfite-Sequenced Libraries

Bisulfite-sequenced reads were filtered by removing adapter sequences and low-quality reads containing more than 50% low-quality bases (quality score < 5). The clean reads were obtained and aligned to the rabbit reference genome (GCF_000003625.3) with using Bisulfite Sequence Mapping Program (BSMAP-2.0) software (http://code.google.com/p/bsmap, accessed on 7 January 2019) in the conditional mode of the map to two forward strands, i.e., BSW (++) and BSC (− +) [37]. The bisulfite conversion rate was calculated and based on the comparison of a small genome without methylation. Here, lambda phage DNA was used as the control group for calculation. The maximum bisulfite conversion rate was got by comparing it with the control genome. 

### 2.5. Analysis of Mean DNA Methylation Levels 

The mean methylation levels were calculated using the bisulfite conversion rate = (100 × Number of CG methylation reads/(Number of CG methylation reads + Number of CG nonmethylation reads)). All coding sequences of genes were divided into seven different transcriptional units regions, which were composed of gene upstream, exon, intron, internal exon, internal intron, last exon, and gene downstream. The upstream region was located at 2kbp before the transcription start site (TSS), and the downstream region was located at 2kbp after the last exon region. DNA methylation levels in these transcriptional units of the genome were calculated.

### 2.6. Screening for Differentially Methylated Regions (DMRs)

Differentially methylated regions (DMRs) were defined as the same DNA region with significant methylation sites differences in the genome of samples between groups. DMRs were evaluated by comparing the same position in genome fragments of each library from two experimental groups. Statistical details were as follows: (1) find a window containing at least 10 CpG (CHG or CHH) at the same location in the genome of samples from the two experimental groups; (2) take 10 CpG as windows and 1 CpG as steps to move forward; (3) calculate the mean methylation levels of the two CG samples within this window (each C site covers at least four supporting methylated reads); (4) determine whether a difference exists between samples through inspection (value difference of methylation level >0.1); and (5) continue to step until no difference. Then, these preselected intervals were combined to obtain the final DMRs.

### 2.7. Differential Alternative Splicing Analysis

Based on the previous comparison with the genome, the replicate Multivariate Analysis of Transcript Splicing (rMATS 4.0.2) software (http://rnaseq-mats.sourceforge.net, accessed on 7 January 2019) was used to analyze the differential expression of five types of alternative splicing and gene statistics between samples of two groups [38]. The parameters of the software were referred to in accordance with the RNASeq−MATS.py−analysis U−t paired−a 8. P and FDR values were used to measure the significance of alternative splicing differences between the two groups. We used FDR ≤ 0.05 as the threshold value to identify differentially spliced genes (DSGs) between the two groups. We also used rMATs to analyze alternative splicing events in a single sample relative to the genome annotation information.

### 2.8. Enrichment Analysis

DMR genes and variable splice genes were analyzed for enrichment analysis using DAVID (https://david.ncifcrf.gov/tools.jsp, accessed on 10 June 2020) and the KEGG database (Kyoto Encyclopedia of Genes and Genomes). R programs and Graphpad Prism 8.0 were used to construct diagrams.

### 2.9. Network Analysis 

The interaction network was constructed using STRING 11.0 (https://version-11-0.string-db.org/, accessed on 9 July 2020) and Cytoscape software 3.7.0 The hub genes were screened for using the Cytohubba program of Cytoscape software 3.7.0. Cytohubba has twelve algorithms < Degree, Edge Percolated Component (EPC), Maximum Neighborhood Component (MNC), Density of Maximum Neighborhood Component (DMNC), Maximal Clique Centrality (MCC) and centralities based on shortest paths, such as Bottleneck (BN), Eccentricity, Closeness, Radiality, Betweenness, and Stress > for searching the hub genes in the network [39].

## 3. Results

### 3.1. Differential Methylation of the Skeletal Muscle Tissue between the Two Groups

Approximately 80% of the reads of the whole-methylome bisulfite sequencing data from skeletal muscle were uniquely mapped to the rabbit reference genome (Table 1). We analyzed the deep sequencing distribution to check the validity of the methylome data of skeletal muscle samples from CON−G and HFD−G (Figure S1). The sequence characteristics of Cytosine (C) were divided into three types: CG, CHG, and CHH (H stands for A or T or C base) [40]. The effective deep sequencing cumulative distribution of C base types based on effective data in CON−G and HFD−G was shown in Figure S2. The effective coverage rates of different C base types in the whole genome indicated that the methylation results were reliable for subsequent studies. 

### 3.2. Distribution Ratios of Methylated C Bases between the Two Groups

The distribution ratios of methylated C bases mCG, mCHG, and mCHH differed among samples of the two groups. The number and proportion of methylated C bases (mCG, mCHG, and mCHH) reflected the characteristics of the genome-wide methylation map in a skeletal muscle tissue sample of rabbits. The composition ratios of mCG, mCHG, and mCHH in the CON−G and HFD−G were shown in Table 2. Proportionally, the proportion of mCG in HFD−G (94.018%) was higher than those in CON−G (93.756%), and the ΔmC proportion of mCG (0.637%) was higher than those of mCHG and mCHH (0.094% and 0.269%, respectively). Results indicated that the higher difference of mCG methylation occurred in the skeletal muscles of obese rabbits after the high-fat induction process. 

### 3.3. Analysis of DNA Methylation Levels and Differentially Methylated Regions (DMRs) between the Two Groups

**Figure 1:**
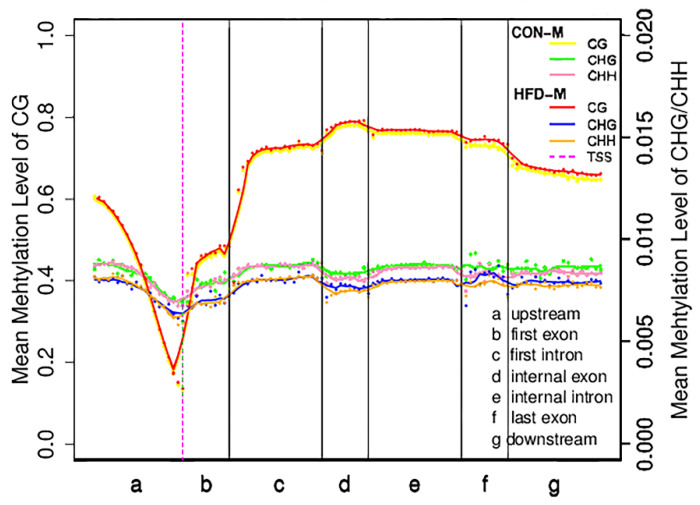
Distribution of mean methylation levels in functional areas of the gene region. This figure represented the methylation levels in the samples of CON-M and HFD-M groups. The entire gene was divided into seven different transcriptional elements in the *X*-axis. The length of each transcriptional element area was divided into an equal number of bin areas containing a certain number of bases. Each dot represented the mean methylation level of a bin region. The curve represented a five-point mean value of methylation level in each bin region. The vertical axis was the mean methylation level (values range from 0 to 1). The purple dotted line was the TSS position (Transcription start site). Different colored lines and dots represented different types of methylation levels between the two groups, respectively.

The distribution characteristics of DNA methylation levels in different functional regions are helpful to understand the role of DNA methylation modifications in different regions at the genome-wide level [42]. To facilitate our understanding of the internal relationship between DNA methylation and gene expression, we calculated the mean methylation levels of the seven different transcriptional units’ regions in all coding sequences of genes (Figure 1). Results showed that the mean CG methylation levels in different regions of the HFD−G were slightly higher than those of the CON−G, while the mean methylation levels of CHG and CHH in the HFD−G were lower than those of the CON−G, suggesting that the mean methylation levels of the three types in different regions of the gene may be closely related to high-fat diet induction. Furthermore, we found that 5390 DMRs were distributed across the 21 pairs of autosomes and 1 pair of sex chromosomes in CON−G and HFD−G with a total length of about 1,570,251 base pairs (Table 3). These results indicated that the high-fat diet could induce the production of evident methylation regions in the skeletal muscles of rabbits.

### 3.4. Methylation of Genes Analysis between the Two Groups

**Figure 2:**
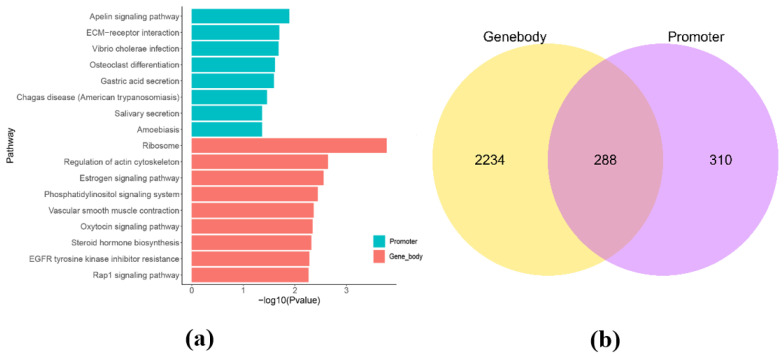
Enrichment analysis of genes methylated in the regions of promoter and gene body between CON−G and HFD−G. (**a**) KEGG enrichment analysis of genes in promoter and gene region, respectively. (**b**) Venn diagram of differential genes methylated in the regions of promoter and gene body from the two groups.

In this study, we identified 599 promoter methylated genes (Demethylated genes: 266; Methylated genes: 333) and 2522 gene body methylated genes (Demethylated genes: 1143; Methylated genes: 1379) by comparing DMRs in the promoter and gene body regions of the genome. To better understand the potential function of these genes, we performed a functional enrichment analysis of methylated genes in these regions. The results of functional enrichment analysis of genes methylated in the regions of promoter region (Figure 2a and Table S1) revealed metabolism-related pathways such as the apelin signaling pathway (ko04371, *n* = 12, *p* = 0.01281), ECM-receptor interaction (ko04512, *n* = 9, *p* = 0.01995), vibrio cholerae infection (ko05110, *n* = 6, *p* = 0.02058), osteoclast differentiation (ko04380, *n* = 11, *p* = 0.02433), and gastric acid secretion (ko04971, *n* = 8, *p* = 0.02546). The results of functional enrichment analysis of genes methylated in the regions of gene body region (Figure 2b) revealed metabolism-related pathways such as ribosome (ko03010, *n* = 15. *p* = 0.0001656), regulation of actin cytoskeleton (ko04810, *n* = 59, *p* = 0.002279), estrogen signaling pathway (ko04915, *n* = 32, *p* = 0.002765), phosphatidylinositol signaling system (ko04070, *n* = 35, *p* = 0.003577), vascular smooth muscle contraction (ko04270, *n* = 38, *p* = 0.004323), oxytocin signaling pathway (ko04921, *n* = 43, *p* = 0.004541), and rap1 signaling pathway (ko04015, *n* = 64, *p* = 0.005431). The Venn diagram showed that 288 genes were found to exist two types of methylation (promoter and gene body; Figure 2c). Based on the intensity of the methylation rate and the function of the genes, 10 methylated genes were screened out from the 288 dual-methylated genes. The 10 methylated genes among the 288 dual-methylated genes for further research were based on their promoter methylation rates at the Table 4.

### 3.5. Analysis of Alternative Splicing between the Two Groups

**Figure 3:**
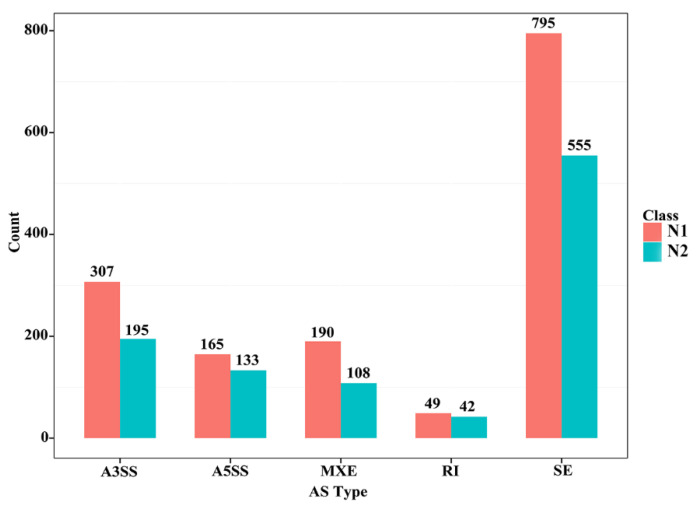
Several different alternative splicing events and unique genes in skeletal muscle between the two groups. N1 and N2 represented the number of alternative splicing events identified in each category and the number of unique genes with such alternative splicing changes, respectively.

Different splicing events enable a gene to produce different types of transcripts; different transcripts can perform different molecular functions. Five types of alternative splicing types were found in the skeletal muscle of CON−G and HFD−G rabbits by comparing mRNA and genome sequencing. These five alternative splicing types were composed of exon skipping (SE), intron retention (RI), alternative 5′ splice site (A5SS), alternative 3′ splice site (A3SS), and mutually exclusive exon (MXE) in Figure 3. Exon skipping had the largest number of splicing events among all five types, with 795 total splicing events in 555 genes. The results demonstrated that a high-fat diet can induce different types of variable shear events in the skeletal muscle tissue of rabbits.

### 3.6. Functional Enrichment Analysis of Genes with Related to ES Alternative Splicing 

To better understand the role of genes related to differentially alternative splicing in the skeletal muscle of CON−G and HFD−G rabbits, we performed a functional enrichment analysis of genes related to ES alternative splicing and differentially methylation by using the online DAVID software. These genes were involved in six pathways (*p* < 0.05, Table 5), i.e., hypertrophic cardiomyopathy (HCM), focal adhesion, regulation of actin cytoskeleton, glycosylphosphatidylinositol (GPI)-anchor biosynthesis, mRNA surveillance pathway, and spliceosome. 

### 3.7. Combined Network Analysis of Exon-Skipping Alternative Splicing and Methylated Genes

**Figure 4:**
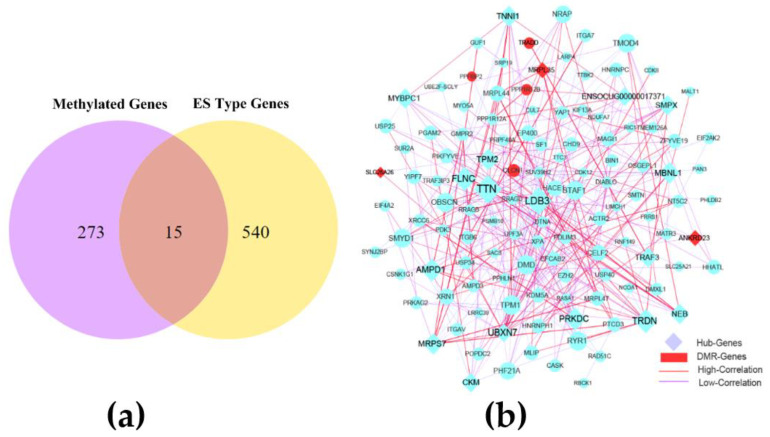
The combined network construction and key genes analysis in skeletal muscle. (**a**) Venn diagram analysis of important genes with two modification modes. (**b**) Interaction network of key genes with exon-skipping alternative splicing and genes in differentially methylated regions (DMR) in skeletal muscle from CON−G and HFD−G rabbits. Hub genes were highly correlated genes within candidate modules.

The combined analysis of 288 genes methylated in promoter and gene body regions and 555 ES alternative splicing genes revealed 15 methylated genes (*ABLIM1*, *N4BP2L2*, *SLC25A26*, *PPP1R12B*, *MRPL35*, *ZBTB20*, *TRADD*, *CLCN1*, *NUFIP1*, *ANKRD23*, *NBAS*, *MACF1*, *PPFIBP2*, *IFT46*, and *MARCHF8*) associated with ES alternative splicing in the skeletal muscle of CON−G and HFD−G rabbits and showed in Figure 4a. Through comparative analysis of the methylation rate and expression level of these genes, we found that the methylated genes did not strongly correlate with their expression levels, suggesting the complex regulation of gene transcription. Subsequently, 555 genes with variable splicing SE and 15 genes with two genetic modifications were used to construct the interaction network. After removing genes with a lower correlation, we set the correlation between the nodes to be higher than 0.4 to construct the network, and showed in Figure S3. Subsequently, the key interaction network between methylated genes and variable shear genes was constructed and showed in Figure 4b. 20 hub genes were identified by using the cytoHubba module of the Cytoscape software in the rabbit skeletal muscle (Table 6). 

## 4. Discussion

A high-fat diet is an important factor that causes body fat deposition and is often used as an inducement condition in studies of energy metabolism and obesity. Compared with vegetable fat, animal fats contain more saturated fatty acids, which are more likely to cause obesity and metabolic disorders [43,44]. Then, we used a high-fat diet containing 10% lard to induce the metabolic characteristics of obesity in rabbits, such as increased subcutaneous and visceral fats, and significant differences in insulin and glucose levels in the obese group [33]. Most studies showed that DNA methylation and the variable shear forms of genes play key roles in the deposition and metabolism of fat in mice [45,46,47]. In this study, we found that the skeletal muscle of obese rabbits displays evident changes in the DNA methylation and alternative splicing landscape as compared with their control counterparts. As differences in methylation patterns between parents and children may lead to epigenetic defects [46,48] and DMRs for various characteristics can be maintained during the parent-progeny inheritance process and help with adaptation to the external environment [47,49], our results may be useful in understanding the potential implications of obesity in epigenetic inheritance. Methylation in the promoter region has a high correlation with the regulation of gene expression, whereas genes with gene body methylation have a complex regulatory relationship with gene expression [50]. Genes with evident differential methylation in the promoter and gene body regions have a remarkable influence on gene expression and genetic effects [51]. Similarly, we found genes with states of hypermethylation and hypomethylation in the promoter and gene body regions of genes in muscle samples from CON−G and HFD−G rabbits. The hypermethylation and hypomethylation of genes may play key roles in the regulation of the proliferation and differentiation of cells, which can immediately affect the downstream of genes to adapt to biological changes [52,53]. Obesity can promote the occurrence of tumors by inhibiting anticancer immune regulatory factors [54]. Dietary intervention is also considered to be one of the important means to suppress obesity-related tumors effectively [55]. In this study, we used a high-fat diet to induce obesity in rabbits and we found that the skeletal muscle of such animals displays evident changes in the DNA methylation and alternative splicing landscape as compared with their control counterparts. We found that *UTP18*, paraneoplastic Ma (*PNMA*) 1, and *SLC25A47* genes have higher methylation in the promoter and gene body regions, suggesting that this methylation may be a protective regulation mechanism for rabbits and deserves further study. UTP18 is a component of the small subunit processome involved in the cleavage of pre-ribosomal RNA to form the 18S ribosomal RNA component of 40S ribosomal subunits [56]. PNMA 1 is a member of the PNMA family, which is closely linked to autoimmunity and neurodegeneration. Studies demonstrated that *PNMA 1* encodes a pro-apoptotic protein in neurons to affect paraneoplastic neurological syndrome and hepatocellular carcinoma progression [57,58], which regulate the growth and development of cancer and tumor cells.

The mammalian target of the rapamycin (mTOR) signaling pathway can regulate muscle protein synthesis and degradation [59]. LAMTOR1 is regarded as a membrane protein, which is specifically localized to the surface of late endosomes/lysosomes and can interact with RagAB/CD GTPases and V-ATPase to activate the mTOR complex 1 (mTORC1) signaling pathway [60]. EIF3 is also a downstream regulator of mTOR, whereas CENPH can indirectly regulate the mTOR signaling pathway [61]. EIF3 and CENPH can maintain cell protein synthesis homeostasis through the mTOR signaling pathway. The knockout of the *eIF3f* gene inhibits mTOR expression, thereby hindering mouse embryonic development and reducing adult skeletal muscle mass [62]. The demethylation of *LAMTOR1*, *CENPH,* and *EIF3J* genes in the promoter region indicates that the mTOR signaling pathway may be activated to regulate the protein metabolism of muscle in obese rabbits. A higher correlation is observed between the methylation ratio and the expression levels of these genes, such as *UTP18*, *PNMA1*, *HOXB5,* and *CENPH*, suggesting special genetic modification modes.

The accumulation of excessive fat in the skeletal muscle resulting from a high-fat diet leads to high differences in energy metabolism, such as decreased insulin sensitivity, abnormal mitochondrial metabolism, and increased inflammatory response levels [63]. The functional enrichment analysis in this study found that the genes methylated in the regions of the promoter and gene body are associated with many metabolism-related signal pathways. The extracellular matrix (ECM)-receptor interaction pathway is involved in adipose differentiation and fat deposition in three fat tissues (i.e., subcutaneous, visceral, and intramuscular adipose tissues) [64,65]. Notably, we also observed the regulation of actin cytoskeleton and glycosylphosphatidylinositol (GPI)-anchor biosynthesis pathway, which is enriched by different ES-type genes. This finding is consistent with the enriched pathway of methylated genes in the gene body region. Studies demonstrated that changes in skeletal muscle metabolic patterns can cause the actin cytoskeleton to rearrange and activate glycolipid signaling pathways [66,67]. These results confirmed that the excessive accumulation of fat in the skeletal muscle affects the functions of glycolipid proteins, skeletal muscle cell development, and inflammatory response.

To understand the relationship between DNA methylation and alternative splicing events, we found 15 genes with existing two genetic modification mechanisms from the Venn diagram. Interestingly, the methylation rates of these genes have a lower correlation with their mRNA expressions levels, such as *IFT46*, *MARCHF8*, *PPFIBP2*, and *ZBTB20*. Then, we speculated that the selection of DNA methylation regions may increase the possibility of the alternative splicing of genes to form various protein functions for regulating the biological process. In addition, gene methylation may affect the expression of tissue-specific splicing variants to form various biological functions. Combined networks were constructed, and the CytoHubba module function of Cytoscape was used to analyze the core gene regulatory network to understand the potential relationship between 15 genes and other SE-type variable splicing genes. A previous study showed that ES events constitute a large proportion of alternative splicing events and are important to the occurrence of biological events [68]. Interestingly, most hub genes with alternative splicing modification were the key nodes in the network, suggesting their association with various biological functions in response to obese metabolism in rabbits. Integrin genes are involved in the HCM signaling pathway, which mediates the transduction of intracellular and extracellular signaling molecules and is closely related to cardiovascular disease and inflammation [69,70]. Abnormalities in integrin genes can cause type 1 diabetes in children [71]. In this study, we found that integrin genes (i.e., *ITGAV*, *ITGB6*, and *ITGA7*) were highly associated with other hub genes in the combined network, such as *GMPR2*, *AMPD3*, and *MRPS7*, suggesting their various roles in the regulation of metabolism in the muscle.

The absorption of dietary fat can increase body energy reserves and change the metabolic pattern in the skeletal muscle tissue. The creatine/creatine phosphate system plays an important role in the process of intramuscular energy metabolism. The creatine kinase (*CKM*) gene encodes a muscle-specific isoenzyme of CKM, which is a key enzyme for skeletal muscle energy metabolism and is also regarded as a marker of muscle development and differentiation in the skeletal muscle. In addition, the CKM protein has a high affinity with saturated and monounsaturated fatty acids with phosphatidic acid, and its expression level is closely related to the formation of various types of intramuscular fatty acids [72]. The diabetes-associated connexin repeat (DARP/ANKRD23) protein is a member of the muscle connexin repeat protein family and can negatively regulate the expression of liver kinase B1 (*LKB1*) in the skeletal muscle, thereby promoting the LKB1/AMPK energy metabolism signaling pathway to maintain a steady glucose state [73]. The expression of mitochondrial proteins can regulate the intramuscular metabolic balance. The SLC25A26 carrier protein can affect RNA stability, protein modification, and mitochondrial gene translation [74]. Furthermore, the DNA-dependent protein kinase gene (*PRKDC*) can affect DNA replication, and its mutations or deletions can lead to immunodeficiency and DNA repair disorders [75]. In the present study, we also found that *CKM*, *ANKRD23*, *SLA25A26*, and *PRKDC* genes were the key nodes that interact with other genes in the network. Particularly, *ANKRD23* and *SLA25A26* genes were demethylated in promoter and gene body regions, and their gene expression levels were increased in obese skeletal muscle tissues, suggesting that their association with the regulation of intramuscular metabolism was induced by a high-fat diet in rabbits.

Ubiquitination modification can mediate the degradation of proteins, which can regulate a variety of cellular activities, such as transcription, DNA damage repair, and immune response. The UBXD7 protein contains an UBX domain, which can bind to multiple ubiquitin ligases for protein degradation [76]. When combined with mitochondrial ubiquitin-protein ligase 1, the UBXD7 protein regulates the level of HIF-1a protein that causes oxidative phosphorylation and changes the level of glycolysis [77]. Mitochondrial ribosomal proteins (MRPs) are synthesized in the cytoplasm and transported to the mitochondria for the translation of mitochondrial proteins [78]. MRPS7 belongs to the MRP family and is transported by nuclear gene-encoded proteins to the mitochondria to participate in the mitochondrial respiratory chain metabolism [79]. In this study, *UBXD7* and *MRPS7* were the core regulation centers in the network. Simultaneously, *MRPL35* was demethylated in the promoter and gene body regions, thereby increasing the gene expression level. Then, we speculated that the mitochondrial metabolism in skeletal muscle was enhanced under high-fat-induced conditions. These genes are worthy of further studies in the regulation of rabbit muscle mitochondrial metabolism.

The excessive accumulation of fat in skeletal muscles affects normal muscle development, changes muscle fiber types, and causes intramuscular metabolic diseases. Mutation in functional sites related to actin factors can cause muscle disease. The titin (*TTN*) and LIM domain binding 3 (*LDB3*) actin genes exhibit alternative splicing in the skeletal muscle. Mutations in *TTN* and *LDB3* genes can cause the abnormal development of the skeletal muscle, leading to muscular dystrophy [80,81,82]. Mutations closely related to muscle development muscleblind-like (MBNL) proteins affect the regulation of alternative RNA splicing for the development of striated muscle after birth, which is closely related to the onset of myotonic dystrophy [83]. Mutations in the actin-binding protein serine C (FLNC) and the small heat shock protein 7 (HSPB7) produce abnormal muscle fibers and damage muscle fiber membranes [84]. Myosin binding protein C1 (MYBPC1) is an abundant skeletal muscle protein and is expressed in slow muscle fibers [85]. Slow- (TNNI1) and fast-twitch (TNNI2) skeletal muscle isoforms are important proteins for the formation of different muscle fibers. These isoforms are located on striated muscle filaments and participate in the inhibition of the calcium-induced myosin ATPase activity, which is closely associated with meat quality [86]. The *AMPD1*, small muscle protein X-linked, and triadin (*TRDN*) genes are closely related to the improvement of meat quality [87,88,89]. In the present study, *TTN*, *LDB3*, *FLNC*, *TRDN*, *AMPD1*, *MBNL1*, *MYBPC1*, and *LOC100349824* were the key nodes in the network and probably influence the formation of skeletal muscle fiber types in obese rabbits. 

In addition, the DNA methylation level of genes has inconsistent changes in the gene promoter and gene body regions, and its mRNA expression level was still expressed in muscle tissues, which may involve a variety of genetic modification mechanisms, such as non-coding RNA, methylated transferase, and histone modifications. Notably, we found and speculated that methylated genes may form an interaction with variable splicing, which can increase the mRNA expression level to influence various functions of the encoding protein. The important modification mechanisms of these genes are worthy of further studies and can improve the meat quality of rabbits by targeted modification techniques. However, this study has the limitations, such as mixed samples, only one library in each group for WGBS, and sequencing methods. The functional verification of genes and networks should be considered in further studies. 

## 5. Conclusions

In this study, we used the data from DNA methylation and alternative splicing analysis to construct the combined interaction network, which showed that *CKM*, *ANKRD23*, *SLA25A26*, and *PRKDC* were mainly involved in metabolism. *UBXD7* and *MRPS7* were involved in ubiquitinated protein degradation pathways, and *TTN*, *LDB3*, *FLNC*, *TRDN*, *AMPD1*, *MBNL1*, *MYBPC1*, and *ENSOCUG00000017371* were involved in muscle development. 

## Figures and Tables

**Table 1:** Whole-methylome bisulfite sequencing data from skeletal muscle in CON−G and HFD−G ^1^.

Group	Clean Reads	Clean Bases (bp)	Mapped Reads	Mapping Rate (%)	Unique Mapped Reads	Unique Mapping Rate (%)	Bisulfite Conversion Rate (%)
CON−G	606,327,246	90,949,086,900	515,160,587	84.96	486,303,133	80.2	99.44
HFD−G	604,790,856	90,718,628,400	514,847,548	85.13	485,505,500	80.28	99.52

^1^ Bisulfite conversion rate = 1 − methylation rate of control DNA.

**Table 2:** Number and proportion of methylated C bases in CON−G and HFD−G ^1^.

	CON−G		HFD−G		HFD−G VS CON−G
	mC Number	Proportion (%)	mC Number	Proportion (%)	ΔmC/All ΔmC Proportion (%)
mCG	51,298,929	93.756	50,997,492	94.018	0.637
mCHG	903,726	1.652	859,466	1.584	0.094
mCHH	2,512,479	4.592	2,385,160	4.397	0.269

^1^ Methylated C (mC) screening method [41]. Binomial distribution test performed for methylated reads and non-methylated reads at C sites. The table shows the number and proportion of methylated reads greater than or equal to their expected value in the binomial distribution over a total effective coverage greater than or equal to 4.

**Table 3:** Number and length of differentially methylated regions (DMR) per chromosome in CON−G and HFD−G.

	chr1	chr2	chr3	chr4	chr5	chr6	chr7	chr8	chr9	chr10
DMR number	1331	1401	1051	776	335	496	617	742	1149	419
DMR length	396,041	421,303	305,229	218,887	96,186	132,605	187,769	219,937	331,992	125,477
	chr11	chr12	chr13	chr14	chr15	chr16	chr17	chr18	chr19	chr20
DMR number	509	892	1538	735	585	736	688	683	697	269
DMR length	154,160	265,709	426,667	221,132	178,538	214,250	194,860	196,669	194,099	74,580
	chr21	chrX	Total							
DMR number	264	476	5390							
DMR length	71,604	156,157	1,570,251							

Note: The number and length of DMR were the total numbers and total length of differential fragments in each chromosome.

**Table 4:** The key methylated genes were selected among the 288 skeletal muscle dual-methylated genes (promoter (P-DMR) and gene body (G-DMR) regions) in rabbits from the two groups.

Description and Genes	Log2Ratio (HFD-M/CON-M)		Means-Gene Expression Levels		Log2FC (HFD-M/CON-M)	Up-Down-Regulation (HFD-M/CON-M)
	P-DMR	G-DMR	CON-M	HFD-M		
Solute carrier family 25 member 47 (*SLC25A47*)	3.036	1.807	40.74781	41.64461	0.031407	Up
Homeobox B5 (*HOXB5*)	−2.907	1.034	7.40671	10.85412	0.551339	Up
Late endosomal/lysosomal adaptor, MAPK, and MTOR activator 3 (*LAMTOR3*)	−2.722	−1.068	207.7547	228.8373	0.139441	Up
Tetratricopeptide repeat domain 29 (*TTC29*)	−2.03	−1.512	0.50368	0.511492	0.022204	Up
Centromere protein H (*CENPH*)	−1.914	−1.256	325.2772	354.271	0.123183	Up
UTP18 small subunit processome component (*UTP18*)	2.773	1.068	202.6769	175.4434	−0.20818	Down
PNMA family member 1 (*PNMA1*)	2.1	1	7.949022	7.626103	−0.05983	Down
Serpin family E member 1 (*SERPINE1*)	−2.138	1.034	313.767	182.1212	−0.78479	Down
Eukaryotic translation initiation factor 3 (*EIF3J*)	−3.524	4.524	426.54	424.0606	−0.00841	Down
Intraflagellar transport 46 (*IFT46*)	−2	−0.979	172.7577	170.0518	−0.02278	Down

**Table 5:** Enriched pathways associated with differentially alternative splicing genes in the skeletal muscle of CON−G and HFD−G rabbits.

KEGG Pathway Terms	Fold Enrichment	Bonferroni	Benjamini	*p* Value	FDR
ocu05410: Hypertrophic cardiomyopathy (HCM)	4.3911	0.1647	0.1798	0.0009	0.1798
ocu05414: Dilated cardiomyopathy	3.6541	0.6843	0.5071	0.0059	0.5071
ocu04510: Focal adhesion	2.5133	0.7829	0.5071	0.0078	0.5071
ocu04810: Regulation of the actin cytoskeleton	2.2706	0.9872	1	0.0223	1
ocu00563: Glycosylphosphatidylinositol (GPI)-anchor biosynthesis	5.9221	0.9963	1	0.0286	1
ocu03015: mRNA surveillance pathway	2.9955	0.999935	1	0.0487	1
ocu03040: Spliceosome	2.3853	0.9999	1	0.0493	1

**Table 6:** Top 20 genes associated with high-fat content in skeletal muscle of rabbits.

Description and Genes	Means-Gene Expression Levels		Log2FC (HFD-M/CON-M)	Up-Down-Regulation (HFD-M/CON-M)	Degree	Type
	CON-M	HFD-M				
UBX domain protein 7 (*UBXN7*)	395.9702	357.9715	−0.14555	Down	28	Hub
Titin (*TTN*)	6,762,662	6,303,461	−0.10145	Down	22	Hub
LIM domain binding 3 (*LDB3*)	28,568.54	26,594.22	−0.10331	Down	20	Hub
Filamin C (*FLNC*)	35,904.61	39,938.6	0.153615	Up	15	Hub
Triadin (*TRDN*)	9918.111	9663.512	−0.03752	Down	15	Hub
Adenosine monophosphate deaminase 1 (*AMPD1*)	17,171.82	14,074.44	−0.28697	Down	13	Hub
Protein kinase, DNA-activated, catalytic subunit (*PRKDC*)	531.1487	669.5437	0.334062	Up	13	Hub
Muscleblind like splicing regulator 1 (*MBNL1*)	13,307.22	12,417.02	−0.09989	Down	11	Hub
Mitochondrial ribosomal protein S7 (*MRPS7*)	2238.874	2278.568	0.025354	Up	11	Hub
Tropomyosin 2 (*TPM2*)	33,609.41	60,754.3	0.854121	Up	11	Hub
Troponin I1, slow skeletal type (*TNNI1*)	6160.928	9336.202	0.599688	Up	10	Hub
Myosin binding protein C1 (*MYBPC1*)	15,887.01	36,243.89	1.189891	Up	9	Hub
Small muscle protein X-linked (*SMPX*)	1575.926	3478.111	1.142104	Up	9	Hub
Nebulin (*NEB*)	472,893.1	393,039.4	−0.26684	Down	9	Hub
Creatine kinase, M-type (*CKM*)	211,998.7	169,559.2	−0.32227	Down	8	Hub
TNF receptor-associated factor 3 (*TRAF3*)	232.8944	210.9551	−0.14274	Down	8	Hub
Myosin light polypeptide 6 (*LOC100349824*)	2817.639	2572.484	−0.13133	Down	7	Hub
Mitochondrial ribosomal protein L35 (*MRPL35*)	341.1524	342.8565	0.007189	Up	5	DMR
Ankyrin repeat domain 23 (*ANKRD23*)	82,064.12	84,664.2	0.045	Up	5	DMR
Solute carrier family 25 member 26 (*SLC25A26*)	178.6897	198.0738	0.148582	Up	2	DMR

Degree was the number of nodes interacting with the selected node, and its size is related to the core degree of this node. Hub genes were highly correlated genes within candidate modules.

## Data Availability

Publicly available datasets were analyzed in this study. This Transcriptome data and methylation data can be respectively found in the SRA database: [PRJNA768644] and [PRJNA768643].

## Supplementary Materials

The following are available online at https://www.mdpi.com/article/10.3390/cimb43030110/s1, **Figure S1:** Sequencing depth distribution. The *X*-axis was sequencing depth, and the *Y*-axis was the percentage of sequencing depth. **Figure S2:** Cumulative distribution of C base sequencing depth. The horizontal axis (*X*-axis) of the graph represented the effective sequencing depth, and the vertical axis (*Y*-axis) represented the genome measurement. The proportion of C bases whose sequence depth was not less than the specific sequencing depth in the whole genome. The sequencing depth of all bases in the whole genome was no less than 0 (the sequencing depth of unmeasured C-bases is 0), and the proportion of C-bases with a depth of no less than 0 in the whole genome is 100%. Therefore, the leftmost part of the figure started from 100 (the highest point on the *Y*-axis). As the depth increased (from left to right on the *X*-axis), there were fewer and fewer C bases that meet the depth requirement, and the proportion of all C bases in the whole genome was smaller until it tends to 0 (the lowest point on the *Y*-axis). **Figure S3:** Combined network of genes with SE type and 13 methylated genes in skeletal muscle from CON−G and HFD−G rabbits. The size of the loop (The bigger circle), the color of the loop (Pink and purple), and the color of the line (Purple) indicated the importance and interaction strength of genes, respectively. **Table S1:** Functional enrichment analysis of methylated genes in promoter and gene body regions.
