# Supplementary material for: Molecular Profiling of DNA Methylation and Alternative Splicing of Genes in Skeletal Muscle of Obese Rabbits
Source: Curr Issues Mol Biol. 2021 Oct 11;43(3):1558–75. doi: 10.3390/cimb43030110 (PMC8929151; doi:10.3390/cimb43030110)
Supplement: Supplementary file 1 [file cimb-43-00110-s001.zip › Supplementary File(s)/Table S1.pdf]

Table S1. Functional enrichment analysis of methylated genes in promoter and gene-body regions.

KEGG Pathway	DEGs annotation in term	DEGs with KEGG annotation	Genes annotation in term	All genes with KEGG annotation	Pvalue	FDR	Enrichment factor	Pathway ID
Promoter								
Apelin signaling pathway	12	493	186	17394	0.01281	0.860436	2.276255	ko04371
ECM-receptor interaction	9	493	137	17394	0.01995	0.860436	2.317792	ko04512
Vibrio cholerae infection	6	493	72	17394	0.02058	0.860436	2.940162	ko05110
Osteoclast differentiation	11	493	185	17394	0.02433	0.860436	2.097846	ko04380
Gastric acid secretion	8	493	120	17394	0.02546	0.860436	2.35213	ko04971
Chagas disease (American trypanosomiasis)	8	493	128	17394	0.03477	0.860436	2.205122	ko05142
Salivary secretion	9	493	149	17394	0.04303	0.860436	2.131124	ko04970
Amoebiasis	9	493	149	17394	0.04303	0.860436	2.131124	ko05146
Gene								
Ribosome	15	2161	297	17394	0.000166	2.58E-02	0.406518	ko03010
Regulation of actin cytoskeleton	59	2161	304	17394	0.002279	8.58E-02	1.562151	ko04810
Estrogen signaling pathway	32	2161	137	17394	0.002765	8.58E-02	1.88007	ko04915
Phosphatidylinositol signaling system	35	2161	158	17394	0.003577	8.58E-02	1.783018	ko04070
Vascular smooth muscle contraction	38	2161	179	17394	0.004323	8.62E-02	1.708737	ko04270
Oxytocin signaling pathway	43	2161	211	17394	0.004541	8.62E-02	1.640328	ko04921
Steroid hormone biosynthesis	7	2161	151	17394	0.004792	8.62E-02	0.373135	ko00140
EGFR tyrosine kinase inhibitor resistance	24	2161	98	17394	0.005223	8.62E-02	1.971196	ko01521
Rap1 signaling pathway	64	2161	350	17394	0.005431	8.62E-02	1.471827	ko04015
