# Supplementary figures and images for: Molecular Profiling of DNA Methylation and Alternative Splicing of Genes in Skeletal Muscle of Obese Rabbits
Source: Curr Issues Mol Biol. 2021 Oct 11;43(3):1558–75. doi: 10.3390/cimb43030110 (PMC8929151; doi:10.3390/cimb43030110)

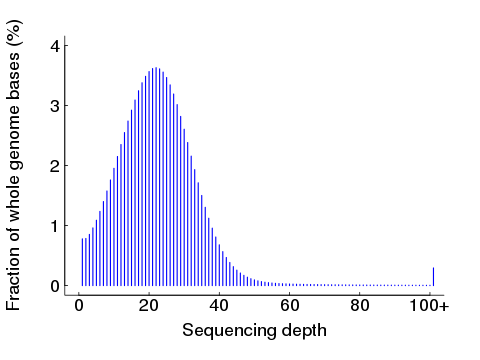

Supplement: Supplementary file 1 [file cimb-43-00110-s001.zip › Supplementary File(s)/Figure S1 CON-M.png]

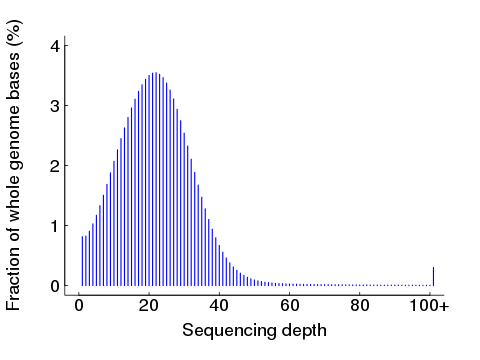

Supplement: Supplementary file 1 [file cimb-43-00110-s001.zip › Supplementary File(s)/Figure S1 HFD-M.png]

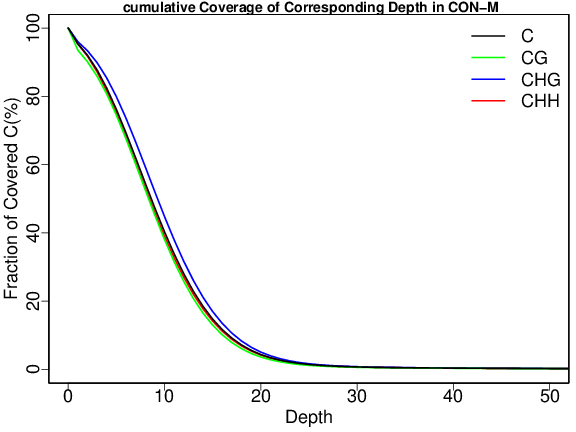

Supplement: Supplementary file 1 [file cimb-43-00110-s001.zip › Supplementary File(s)/Figure S2 CON-M.png]

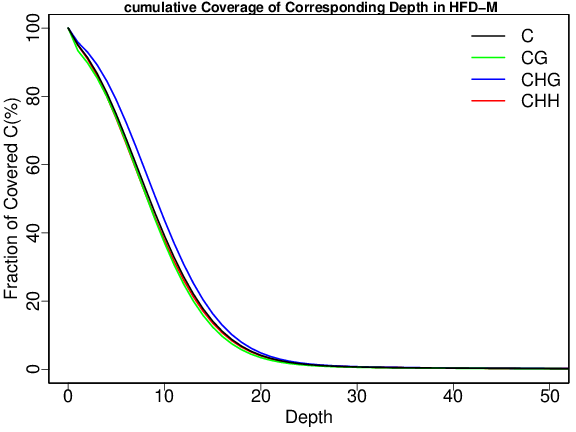

Supplement: Supplementary file 1 [file cimb-43-00110-s001.zip › Supplementary File(s)/Figure S2 HFD-M.png]

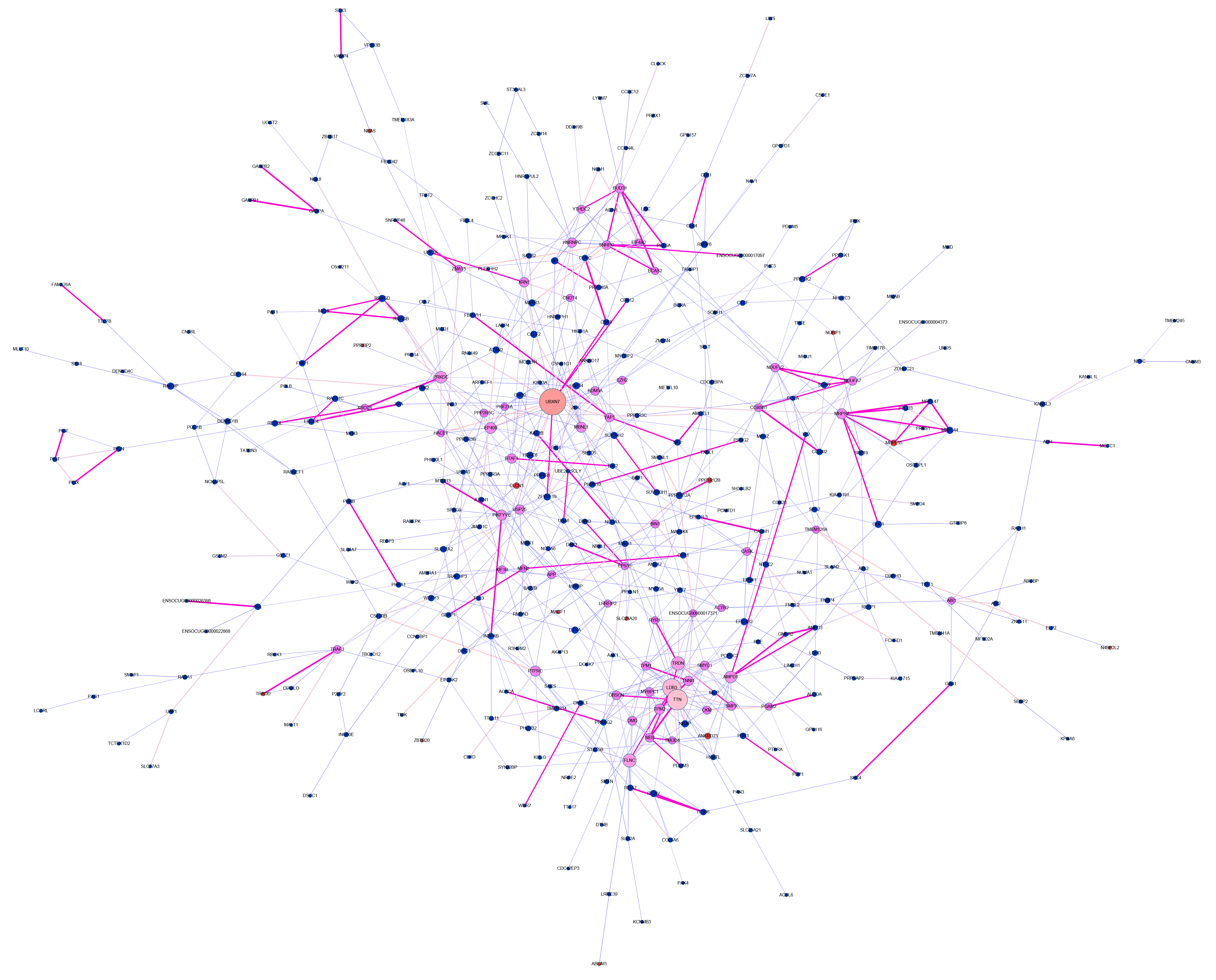

Supplement: Supplementary file 1 [file cimb-43-00110-s001.zip › Supplementary File(s)/Figure S3.png]
